# Persistent Calcium Inadequacy in Korean Adults over 20 Years: Analysis of the 1998–2018 Korea National Health and Nutrition Examination Survey

**DOI:** 10.3390/foods13223568

**Published:** 2024-11-07

**Authors:** Seyoung Ju, Yongseok Kwon, Kyung-Jin Yeum

**Affiliations:** 1 Department of Food and Nutrition, College of Biomedical and Health Science, Konkuk University, Chungju-si 27478, Chungchungbuk-do, Republic of Korea; syoungju86@kku.ac.kr; 2 National Institute of Agricultural Sciences, 166 Nongsaengmyeong-ro, Jeonju 55365, Jeonbuk-do, Republic of Korea; selenium2012@korea.kr

**Keywords:** calcium intake, osteoporosis, elderly, national survey

## Abstract

Calcium inadequacy in Asian populations has been well documented, but whether it has improved over time remains uncertain. We analyzed dietary calcium intake and its association with osteoporosis prevalence over a 20-year period in 48,653 adults (21,932 men and 26,721 women, aged 19 years and older) in Korea, using data from the first to the seventh Korea National Health and Nutrition Examination Survey (1998–2018). Over the past 20 years, Korean adults consistently fell short of the recommended dietary calcium intake, with women and older adults particularly affected, typically consuming only 40–80% of the recommended levels. The 30–49 age group had the highest calcium intake (497–568 mg/day), while those aged 75+ had the lowest (319–457 mg/day). A significant inverse relationship was found between calcium intake and osteoporosis risk, with lower calcium intake associated with higher odds of developing osteoporosis, as determined by both physician diagnoses and bone mineral density measurements (*p* < 0.001). Notably, over the past 20 years, 68–70% of dietary calcium consistently came from plant-based foods. This study strongly emphasizes the urgent need to enhance calcium-rich food availability and implement targeted interventions to increase calcium intake among those most affected by inadequacy, particularly the elderly and women. Further research with recent data would be valuable for understanding current intake levels and evolving nutritional needs.

## 1. Introduction

Calcium is a critical element for bone structure, and its adequate intake is recommended for maintaining general bone health [1]. Despite this, calcium deficiency remains a global concern, with Asian populations particularly at high risk [2]. The average calcium intake among adults in these regions typically ranges from 300 to 600 mg/day [3], which is significantly below the recommended dietary intake of 650–750 mg/day in Japan [4], 700–800 mg/day in Korea [5], and 800–1000 mg/day in China [6]. These recommendations are still often unmet in many Asian populations, despite being lower than those in the US and European countries, where the recommended intake is 950–1200 mg/day [7,8].

The primary dietary sources of calcium in Asian populations, such as vegetables, legumes, and cereals, may not be optimal for calcium absorption due to the presence of phytates and oxalates [9]. For example, the 2015 China Nutritional Transition Cohort Study [10] reported that over 60% of dietary calcium in Chinese diets came from these plant-based sources, with dairy products contributing only 6.7%. This reliance on less bioavailable calcium sources may necessitate higher calcium intake to meet physiological needs, despite the generally smaller body sizes in these populations.

The insufficient intake of calcium, coupled with less favorable sources of dietary calcium, has been linked to a high prevalence of osteoporosis, particularly among postmenopausal women [11,12]. There is also a clear association between low calcium intake and an increased risk of osteoporotic fractures [13,14]. Despite awareness of these risks, calcium intake in some Asian populations, such as Japan, has continued to decline, reaching approximately 480 mg/day by 2016 [15].

Given the alarming trends in calcium intake and the significant health risks associated with calcium deficiency, it is crucial to investigate long-term changes in calcium intake, particularly in populations at high risk of deficiency. Understanding these trends and their implications is vital for developing effective public health strategies to address calcium deficiency and its related health risks. Therefore, the current study evaluated dietary calcium intake among Korean adults over the past 20 years (1998–2018) by analyzing comprehensive data from the Korea National Health and Nutrition Examination Survey (KNHANES I to VII). Additionally, this study examined the major dietary sources of calcium and explored the relationship between calcium intake and the prevalence of osteoporosis in Korea.

## 2. Materials and Methods

### 2.1. Participants

The Korea National Health and Nutrition Examination Survey (KNHANES), initiated in 1998, is a national cross-sectional survey designed to assess the health and nutritional status of Koreans across 600 regions nationwide. Each year, trained personnel, including doctors and nutritionists, collect health and nutritional data from approximately 10,000 individuals. KNHANES I and II were conducted between November and December of 1998 and 2001, respectively. The third KNHANES was carried out between April and June of 2005, and KNHANES IV-1 took place between July and December of 2007. Since 2008, starting with KNHANES IV-2, the national survey has been conducted year-round. The data collection procedures used in this study were consistently followed throughout all phases, wherever possible. The standardized protocols for measurement, participant interviews, and data gathering were largely maintained across the study period to ensure the uniformity and reliability of the data. Any necessary adjustments to the procedures were made as required, and deviations, if any, have been specified where applicable.

**Figure 1:**
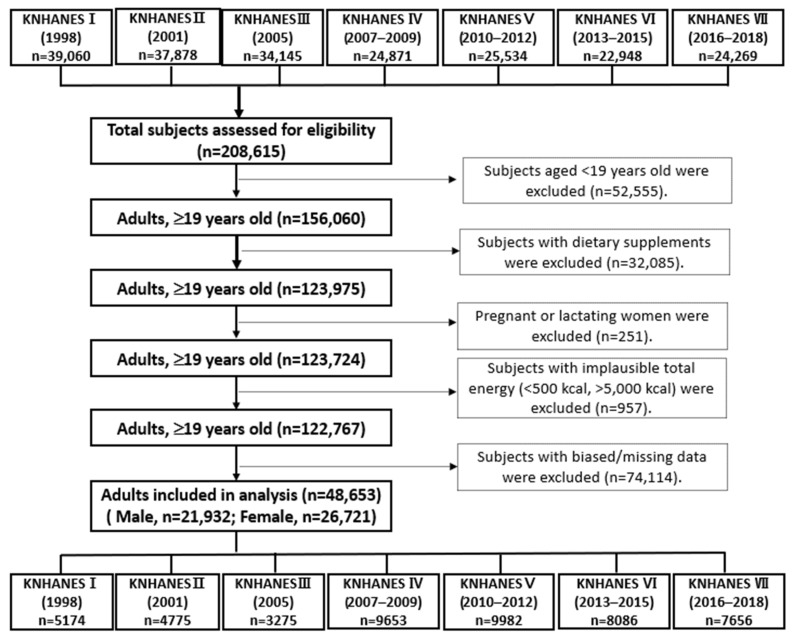
Flow diagram of subject inclusion and exclusion in the Korea National Health and Nutrition Examination Surveys (KNHNES I–VII, 1998–2018).

Data from the first (I, 1998) to the seventh (VII, 2016–2018) KNHANES, which included health examinations and nutrition surveys, were utilized in this cross-sectional analysis. From an initial total of 208,615 men and women, exclusions were made for 52,555 subjects under the age of 19, 32,085 subjects who used dietary supplements, 252 pregnant or lactating women, 957 subjects with implausible energy intakes (<500 kcal or >5000 kcal), and 74,114 subjects with biased or missing data. This resulted in a final sample of 21,932 men and 26,721 women being included in the analysis, as shown in Figure 1. These subjects were distributed as follows: 5174 from KNHANES I, 4775 from KNHANES II, 3275 from KNHANES III, 9653 from KNHANES IV, 9982 from KNHANES V, 8086 from KNHANES VI, and 7656 from KNHANES VII. The KNHANES was approved by the Institutional Review Board (IRB) of the Korea Centers for Disease Control and Prevention, with the following IRB approval numbers: 2007-02CON-04-P, 2008-04EXP-01-C, 2009-01CON-03-2C, 2010-02CON-21-C, 2011-02CON-06-C, 2012-01EXP-01-2C, 2013-07CON-03-4C, 2013-12EXP-03-5C, and 2018-01-03-P-A. The KNHANESs conducted from 2015 to 2017 were carried out without review by the Research Ethics Committee in accordance with Article 2, No. 1 of the Bioethics Act and Article 2, No. 2 of its Enforcement Rule. The Research Ethics Committee review process resumed in 2018 due to the collection of human materials and the provision of raw data to third parties.

### 2.2. Measurements

#### 2.2.1. Health Examination Survey

Health examinations were conducted by trained staff members, including physicians, at a mobile examination center. The health examination survey collected detailed information on socioeconomic status, health behaviors, quality of life, bone density, and other health indicators. Bone mineral density (BMD, g/cm^2^) was measured at the lumbar spine (L1–4) and femoral neck using dual-energy X-ray absorptiometry (DXA) with a DISCOVERY-W fan beam densitometer (Hologic Inc., Marlborough, MA, USA), with coefficients of variation of 1.9% and 2.5%, respectively.

#### 2.2.2. Nutrition Survey

The nutrition survey, which included assessments of dietary behavior, dietary supplement use, food security, food frequency, and food and dietary intake, was conducted through face-to-face interviews in each subject’s home by a trained dietician. Typically, dieticians visited participants’ homes for the nutrition survey about one week after the health examination survey. Nutrient intakes, including total calorie and calcium intakes, were assessed using a 24 h dietary recall questionnaire. The results were calculated using the Food Composition Table developed by the National Rural Resources Development Institute (9th revision). It should be noted that the contents of dietary supplements were not documented in the KNHANES. In this study, the food items were categorized into sixteen food groups: (1) cereals and grain products, (2) potatoes and starches, (3) sugars and sweets, (4) legumes and their products, (5) seeds and nuts, (6) vegetables, (7) mushrooms, (8) fruits, (9) seaweeds, (10) plant oils and fats, (11) beverages, and (12) other plant foods for plant-based foods; (13) meat, poultry, and their products, (14) milk and dairy products, (15) animal oils and fats, and (16) other animal foods for animal-based foods.

### 2.3. Statistics

The Korea National Health and Nutrition Examination Survey (KNHANES) is a national surveillance system that provides nationally representative estimates of the Korean population using a multistage, stratified, cluster sampling design. The statistical analyses in this study were conducted by incorporating stratification, clustering, and sample weight variables, using SAS version 9.4 statistical software (SAS Institute, Cary, NC, USA). The general characteristics of the study population, including age, height, weight, body mass index, and daily nutrient intakes, such as dietary calcium intake, were expressed as means and standard errors using descriptive analysis, and significant differences were verified by using the SURVEYREG procedure. Categorical variables, such as residential area and the prevalence of osteoporosis by year, were expressed as frequency (n) and weighted percentage (%) using frequency analysis, with significant differences analyzed by the χ^2^ test in complex sample analysis. Calcium intakes by sex were adjusted for age and energy intake, while calcium intakes by age group were adjusted for sex and energy intake. For the overall population, calcium intakes were adjusted for age, sex, and energy intake.

The correlation between calcium intake and osteoporosis was assessed using logistic regression analysis, where quintile groups (Q1, Q2, Q3, Q4, and Q5) of calcium intake served as the independent variable and osteoporosis status (osteoporosis, 1; normal, 0) served as the dependent variable, utilizing the SURVEYLOGISTIC procedure. Calcium intakes were divided into quintiles, with the lowest quintile (lowest calcium consumption) used as the reference category. The results are presented as odds ratios (ORs) with 95% confidence intervals (CIs). Logistic regression analysis was conducted in four models: without adjustment (model 1), adjusted for age (model 2), adjusted for age and BMI (model 3), and adjusted for age, BMI, physical activity, and smoking (model 4).

## 3. Results

### 3.1. General Characteristics of the Study Participants

The general characteristics of the study participants from KNHANES I (1998) to KNHANES VII (2016–2018) are presented in Table 1. Over the past 20 years, average calorie intake ranged from 1963 to 2045 kcal/day. The contribution of carbohydrates to total calorie intake decreased from 67.82% in KNHANES I (1998) to 65.70% in KNHANES VII (2016–2018). The contribution of protein fluctuated slightly across the phases, ranging from 13.69% to 15.36%. Meanwhile, the contribution of fats to total calorie intake increased from 16.82% in KNHANES I (1998) to 19.84% in KNHANES VII (2016–2018).

### 3.2. Dietary Calcium Intakes of Korean Adults from 1998 to 2018

**Figure 2:**
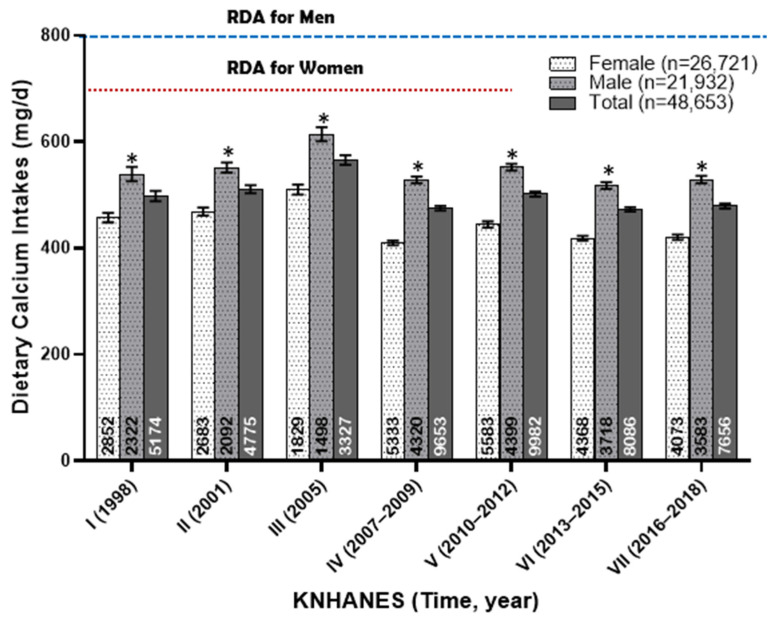
Dietary calcium intake (mg/day) of Korean adults from 1998 to 2018. The annotations at the bottom of each bar represent the number of subjects in each group. An asterisk indicates a significant difference between gender groups at the α = 0.05 level, as determined by the *t*-test.

The dietary calcium intakes (mg/day) of Korean adults from 1998 to 2018 are shown in Figure 2. Over this 20-year period, men had an average dietary calcium intake ranging from 517 to 551 mg/day, while women had a significantly lower intake, ranging from 409 to 468 mg/day. Notably, during KNHANES III, which was conducted over a limited period from April to June in 2005, the average calcium intake temporarily exceeded previous levels, with men consuming 614 mg/day and women 510 mg/day. However, this brief increase did not alter the overall trend, which reveals a consistent insufficiency in dietary calcium intake among Korean adults.

### 3.3. Dietary Calcium Intakes of Korean Adults by Age Group from 1998 to 2018

**Figure 3:**
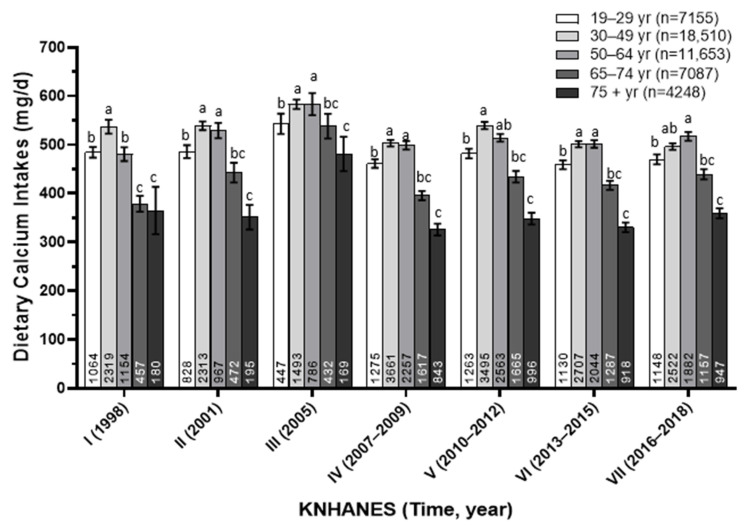
Dietary calcium intake (mg/day) of Korean adults according to age groups from 1998 to 2018. The annotations at the bottom of each bar represent the number of subjects in each group. Different superscript letters indicate significant differences among groups at the α = 0.05 level, as determined by Tukey’s multiple range test.

The dietary calcium intakes (mg/day) of Korean adults were analyzed by age groups over a 20-year period from 1998 to 2018, as shown in Figure 3. Among the different age groups, individuals aged 30–49 consistently had the highest calcium intake, ranging from 497 to 568 mg/day. However, as age increased, calcium intake significantly decreased, with those aged 65–74 consuming between 380 and 514 mg/day. Particularly concerning is the intake among adults aged 75 and older, where calcium intake averaged only 319–457 mg/day, falling well below recommended levels. Even among the younger adults aged 19–29 years, calcium intake was relatively low, generally staying below 500 mg/day, except in 2005. Overall, these findings highlight a consistent insufficiency in dietary calcium intake across all age groups, with older adults, especially those over 75 years, being the most affected.

### 3.4. Dietary Calcium Intakes of Korean Osteoporotic and Normal Adults from 1998 to 2018

**Figure 4:**
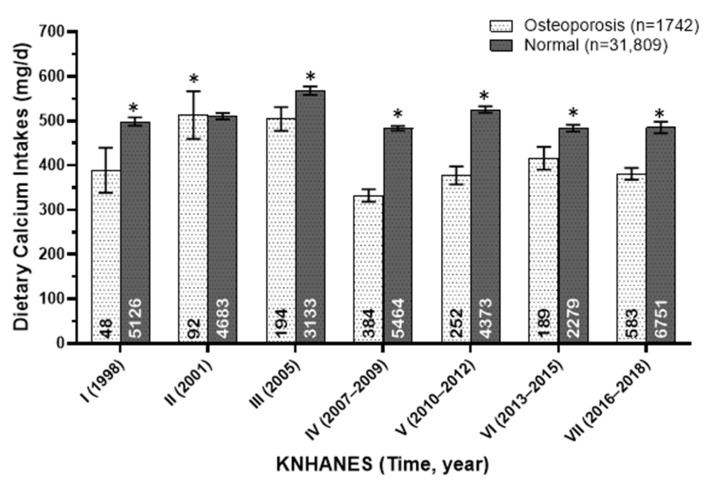
Dietary calcium intakes (mg/day) of osteoporotic and normal adults in Korea from 1998 to 2018. The annotations at the bottom of each bar represent the number of subjects in each group. An asterisk indicates a significant difference between gender groups at the α = 0.05 level, as determined by the *t*-test.

The dietary calcium intakes (mg/day) of osteoporotic and normal adults in Korea from 1998 to 2018 are presented in Figure 4, with osteoporosis including both physician-diagnosed cases and those identified through bone mineral density measurements. Over the past 20 years, calcium intake was consistently and significantly lower in individuals with osteoporosis compared to those without the condition.

### 3.5. Dietary Calcium Sources of Korean Adults from 1998 to 2018

**Figure 5:**
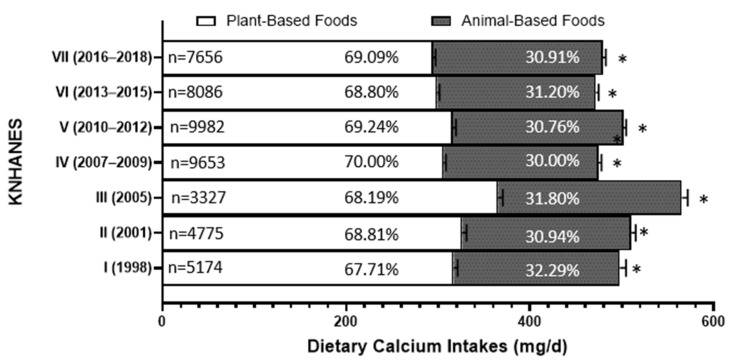
The percentage of total dietary calcium intake from animal-based sources and plant-based sources from 1998 to 2018. An asterisk indicates a significant difference between gender groups at the α = 0.05 level, as determined by the χ^2^-test.

Over the past 20 years, approximately 68–70% of dietary calcium intake for Koreans has consistently come from plant-based foods, while 30–32% has been derived from animal-based foods, as presented in Figure 5. This indicates that more than two-thirds of the dietary calcium sources for Koreans have continuously been plant-based.

**Figure 6:**
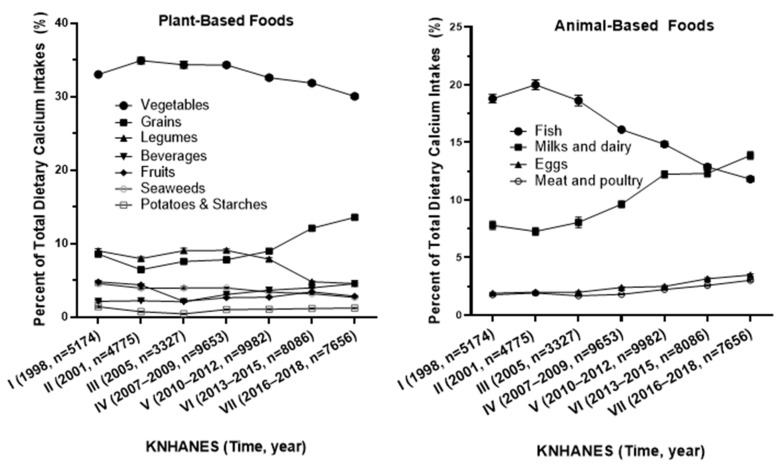
The percentage of dietary calcium intake by food group relative to total dietary calcium intake (%) from 1998 to 2018.

Among the plant-based foods, vegetables were the predominant source of calcium, contributing 30.05% to 34.91% of total calcium intake, followed by smaller contributions from grains, legumes, and seaweed, as presented in Figure 6. In the animal-based food group, the contribution of calcium from fish and shellfish gradually decreased from 20% (KNHANES II) to 11.81% (KNHANES VII). Conversely, calcium intake from milk and dairy products increased from 7.27% of total calcium intake in 1998 (KNHANES I) to 13.86% in 2016–2018 (KNHANES VII). Additionally, the contributions from eggs and meat, poultry, and their products also gradually rose, from less than 2% of total dietary calcium intake in the first KNHANES (1998) to over 5% in the seventh KNHANES (2016–2018).

### 3.6. Association Between Dietary Calcium Intake and Osteoporosis

Table 2 presents the association between dietary calcium intake and osteoporosis as determined by a physician for the entire study population. In Table 2, when participants were classified into five groups based on their calcium intake levels, the odds ratios (ORs) for osteoporosis were significantly lower in the lower quintiles, indicating a strong inverse relationship between calcium intake and the risk of osteoporosis.

Table 3 shows the association between dietary calcium intake and osteoporosis as determined by bone mineral density among the measured subjects. The bone mineral density in the KNHANES was measured using DXA between the years 2008 and 2011, which explains the smaller number of subjects in this table. When osteoporosis was determined by bone mineral density, a strong inverse relationship between calcium intake and osteoporosis risk was observed in the overall analysis without adjustments. This association remained significant when analyzed separately for men and women, despite the reduced sample size. For men, significance was maintained in model 2 after adjusting for age. For women, the association showed strong significance in model 2 and persisted in model 3, even after adjusting for both age and BMI.

## 4. Discussion

This study highlights the severe and persistent calcium inadequacy among Korean adults over the past 20 years, with intake consistently well below recommended levels and predominantly sourced from plant-based foods. This inadequacy is particularly severe among the elderly and women, underscoring the need for targeted nutritional interventions.

Over the observed period in this study, dietary calcium intake among Korean adults has remained largely unchanged, with men rarely exceeding 600 mg/day and women consistently falling below 500 mg/day, which is significantly lower compared to the U.S. trend of 780 to 890 mg/day between 1999 and 2020 [16]. This persistent insufficiency underscores a significant inadequacy in dietary calcium intake, a pattern also observed in other Asian populations, such as Chinese [17], Japanese [18], and Indian [19] individuals. This study found that, for the past two decades, over two-thirds of dietary calcium in the Korean diet has consistently come from vegetables, which have lower absorption rates due to naturally occurring substances, such as oxalate, phytate, and tannin. These compounds, commonly found in many green leafy vegetables, can bind to calcium, making it insoluble and unavailable for absorption in the intestines [9]. In contrast, the proportion of calcium intake from vegetables in the U.S. diet was significantly lower, accounting for 7.03% in 1999–2000 and 6.09% in 2017–2020 [16]. Although vegetables do contain calcium, their lower absorption rates may worsen calcium deficiency, potentially impacting the skeletal health of populations that primarily rely on vegetables as their main source of calcium. This underscores the critical need to promote the consumption of calcium-rich foods with higher absorption rates or to increase the intake of traditional calcium sources. The consistent calcium inadequacy among Korean adults is particularly concerning, as it can lead to decreased bone mineral density and an increased risk of osteoporosis. This study, along with previous research [20], has demonstrated that inadequate calcium intake is closely linked to a higher risk of osteoporosis, highlighting the importance of increasing calcium intake, especially among women, to maintain bone health.

Furthermore, our findings indicate that calcium inadequacy is particularly severe among the elderly. With Korea’s rapidly aging population, the likelihood of bone-related diseases, such as hip fractures, is expected to increase significantly, as suggested by a recent study by Yang et al. [21]. Factors contributing to this inadequacy in the elderly may include dietary habits, economic constraints, and physical limitations that lead to an imbalanced diet. A significant issue contributing to calcium intake inadequacy among Koreans is that over 90% of the population carries the lactose non-persistence gene [22], leading to a low preference for and intake of dairy products, which are major and highly bioavailable sources of calcium for adults. In this study, the intake of calcium from milk and dairy products in Koreans gradually increased from 7.27% of total dietary calcium in 1998 (KNHANES I) to 13.86% in KNHANES VII (2016–2018). In contrast, in the United States, although the percentage of calcium obtained from milk decreased from 33.56% in 1999–2000 to 24.55% in 2017–2020, it still constituted a significant portion of dietary calcium [16]. Women who consume more milk have been reported to have higher bone mineral density in the femoral neck [23], and it is particularly noteworthy that a high habitual milk intake, unlike yogurt or cheese, is associated with a decreased risk of osteoporotic fractures in postmenopausal Japanese women [24], highlighting the beneficial impact of the dairy matrix effect on bone health, as emphasized in recent study [25]. The prevalence of osteoporosis varies significantly across countries with different levels of calcium and dairy intake. According to a recent systematic review of the Asia–Pacific region [26], osteoporosis prevalence in women aged 50 and above is reported to be 8.8–10% in Australia, where dairy and calcium intake is higher, compared to 45% in Korea, where intake is lower. This substantial difference likely reflects lower calcium and dairy consumption in the Korean population, underscoring the critical role of these nutrients in maintaining bone health. Further supporting this, a cohort study from China [27] has demonstrated a strong association between dairy consumption and reduced fracture risk, emphasizing the protective effects of calcium and dairy intake on bone density. Another systematic review [28] corroborates the beneficial impact of dairy consumption in this age group, showing that higher dairy intake can significantly improve bone health and reduce osteoporosis risk. Therefore, it is crucial to identify calcium sources that are both bioavailable and sustainable, similar to milk, for populations who either do not prefer or have difficulty consuming milk. Our recent study, demonstrating the beneficial effects of the long-term consumption of HMR fortified with eggshells on preventing bone mineral density loss in the femoral neck of postmenopausal women [29], suggests a viable alternative source of calcium.

In addition to low calcium intake, vitamin D status among Koreans is also inadequate. Our recent intervention study found that participants had daily vitamin D intake below 5 micrograms [29], reflecting insufficient intake levels. This aligns with previous reports indicating that both calcium and vitamin D deficiencies are prevalent in the Korean population [30]. These two nutrients play critical roles in bone health, as adequate vitamin D is necessary for calcium absorption and optimal skeletal function [31].

Therefore, when discussing the benefits of calcium-fortified products, it is essential to also consider vitamin D supplementation. Ensuring the adequate intake of both calcium and vitamin D is crucial for preventing bone-related health issues, particularly in populations like Korea, where the intake of both nutrients tends to be low.

In this study, dietary intake was assessed using a single 24 h recall. While the 24 h recall method is a widely accepted tool for dietary assessment [32], it has certain limitations. Specifically, a single 24 h recall only captures dietary intake for one day, which may not reflect an individual’s habitual diet. This method is also prone to day-to-day variability and may miss foods that are consumed less frequently. However, we believe that certain measures in our study help mitigate these limitations. First, the data collection was conducted year-round, which accounts for seasonal variations in food intake, thus reducing the concern regarding potential seasonal bias. Furthermore, the large sample size of 48,653 subjects and the 20-year study period enhance the representativeness of the data, allowing for a broader and more accurate reflection of the population’s dietary patterns.

Another limitation of this study is that it only includes data from 1998 to 2018, prior to the COVID-19 pandemic, thus excluding the most recent KNHANES data. Despite this, the trends observed over the past 20 years have been persistent, suggesting that similar patterns likely continue today. Given Korea’s rapidly aging population, it is reasonable to assume that the prevalence of calcium deficiency and related osteoporosis risks may be even more pronounced in recent years. Additionally, findings from our recent intervention study [29] appear to align with these results, indicating that calcium intake among participants remains low, which reinforces the ongoing relevance of these findings for the current Korean population.

## 5. Conclusions

This study demonstrates that calcium intake among Korean adults has consistently been inadequate, with the issue being more severe in older adults due to both insufficient intake and reliance on sources with low absorption efficiency. To address this, strategies should focus on increasing the proportion of calcium-rich foods with higher absorption rates in the diet, such as dairy products, and developing fortified foods that are easily accessible to the elderly. Additionally, there is a pressing need for enhanced national nutrition policies and public health campaigns to educate specific age groups on the importance of dietary calcium. These targeted interventions are crucial to prevent health problems associated with calcium deficiency, particularly among the elderly and women, who are the most vulnerable to its effects. While this study underscores persistent calcium deficiency trends in Korea up to 2018, recent findings indicate that dietary intake may still not meet recommended levels. Given these continued concerns, we recommend an updated assessment of calcium intake in Korea to better understand current needs and guide future intervention strategies effectively.

## Figures and Tables

**Table 1:** General characteristics of study participants from KNHANES I (1998) to KNHANES VII (2016–2018).

	Korea National Health and Nutrition Examination Survey (KNHANES)						
Variable	I (1998) n = 5174	II (2001) n = 4775	III (2005) n = 3327	IV (2007–2009) n = 9653	V (2010–2012) n = 9982	VI (2013–2015) n = 8086	VII (2016–2018) n = 7656
Male/female (n)	2322/2852	2092/2683	1498/1829	4320/5333	4399/5583	3718/4368	3583/4073
Average age	40.57 ^a^ ± 0.37	41.59 ^b^ ± 0.36	43.09 ^c^ ± 0.43	44.06 ^cd^ ± 0.29	44.60 ^d^ ± 0.287	45.20 ^de^ ± 0.30	45.71 ^e^ ± 0.33
Height (cm)	162.99 ^c^ ± 0.18	163.5 ^bc^ ± 0.16	163.61 ^bc^ ± 0.21	164.17 ^b^ ± 0.13	164.35 ^b^ ± 0.14	164.87 ^ab^ ± 0.14	165.15 ^a^ ± 0.14
Weight (kg)	61.46 ^d^ ± 0.20	62.58 ^c^ ± 0.18	63.51 ^bc^ ± 0.26	64.27 ^b^ ± 0.16	64.24 ^b^ ± 0.19	65.35 ^a^ ± 0.18	65.87 ^a^ ± 0.20
BMI (kg/m^2^)	23.08 ^cd^ ± 0.06	23.34 ^c^ ± 0.06	23.65 ^bc^ ± 0.07	23.75 ^b^ ± 0.05	23.68 ^b^ ± 0.05	23.93 ^ab^ ± 0.05	24.04 ^a^± 0.06
Daily Nutrient Intake							
Energy (kcal)	1968.0 ^ab^ ± 19.52	1963.5 ^b^ ± 18.66	2049.7 ^a^ ± 20.99	1906.8 ^c^ ± 11.25	2045.7 ^a^ ± 13.19	2049.6 ^a^ ± 12.18	1970.7 ^b^ ± 13.46
Carbohydrate (g)	322.16 ^a^ ± 3.18	310.67 ^ab^ ± 2.94	314.60 ^ab^ ± 3.16	305.81 ^b^ ± 1.69	322.66 ^a^ ± 1.95	309.44 ^ab^ ± 1.76	295.06 ^c^ ± 1.81
Protein (g)	75.82 ^a^ ± 1.09	73.96 ^a^ ± 0.84	78.04 ^a^ ± 1.04	67.68 ^b^ ± 0.51	73.46 ^b^ ± 0.61	70.99 ^b^ ± 0.57	71.37 ^b^ ± 0.58
Fat (g)	38.99 ^b^ ± 0.72	40.25 ^b^ ± 0.71	43.89 ^a^ ± 0.89	37.34 ^b^ ± 0.44	43.14 ^ab^ ± 0.52	45.29 ^a^ ± 0.52	45.43 ^a^ ± 0.55
Ca (mg)	497.78 ^b^ ± 9.45	510.57 ^ab^ ± 7.44	565.40 ^a^ ± 9.19	474.66 ^bc^ ± 4.29	501.57 ^ab^ ± 4.84	472.76 ^c^ ± 4.11	493.26 ^ab^ ± 4.65
Ratio (%)							
Carbohydrate	67.82 ^ab^ ± 0.29	67.83 ^ab^ ± 0.24	66.55 ^b^ ± 0.28	69.07 ^a^ ± 0.17	67.79 ^ab^ ± 0.18	67.41 ^ab^ ± 0.18	65.70 ^c^ ± 0.19
Protein	15.36 ^a^ ± 0.21	14.86 ^b^ ± 0.08	15.15 ^ab^ ± 0.11	14.13 ^cd^ ± 0.06	14.20 ^c^ ± 0.06	13.69 ^d^ ± 0.06	14.46 ^c^ ± 0.07
Fat	16.82 ^d^ ± 0.22	17.31 ^c^ ± 0.21	18.30 ^b^ ± 0.25	16.80 ^d^ ± 0.14	18.01 ^bc^ ± 0.14	18.90 ^ab^ ± 0.15	19.84 ^a^ ± 0.16

*p*-value (*p* < 0.001) via one-way ANOVA from the SURVEYREG procedure of SAS. Different superscript letters mean significant difference among groups at the α = 0.05 level via Scheffe’s multiple range comparison. Data are expressed as mean ± standard error.

**Table 2:** Association between calcium intake quintiles and the prevalence of physician-diagnosed osteoporosis.

	Q1	Q2	Q3	Q4	Q5	*p* for Trend
Total	n = 7459	n = 7460	n = 7459	n = 7460	n = 7459	
Age (mean, SE)	44.91 ± 0.42	43.65 ± 0.34	42.84 ± 0.31	42.31 ± 0.30	42.42 ± 0.30	<0.0001 (−)
Ca Intake (mg/day)						
Mean, SE	203.15 ± 1.24	344.89 ± 0.72	465.96 ± 0.76	625.30 ± 1.20	1041.39 ± 8.16	<0.0001 (+)
Median, SE	212.80 ± 2.12	345.43 ± 1.27	463.53 ± 1.15	621.03 ± 2.07	929.67 ± 5.94	-
Intake range	5.80–241.89	241.90–353.05	353.11–477.36	477.37–668.24	668.25–11,786.0	-
Prevalence of Osteoporosis						
Normal, n (%)	6776 (93.23)	7039 (96.07)	7092 (96.89)	7210 (97.92)	7196 (97.70)	<0.0001 ^1)^
Osteoporosis, n (%)	683 (6.77)	421 (3.93)	367 (3.11)	250 (2.08)	263 (2.30)	
Odds Ratio (95% Confidence Interval)						
Model 1	1	0.59 (0.39–0.78)	0.52 (0.31–0.73)	0.32 (0.30–0.34)	0.31 (0.29–0.51)	<0.0001 (−)
Model 2	1	0.92 (0.91–1.13)	0.96 (0.87–1.00)	0.81 (0.65–0.95)	1.00 (0.72–1.23)	0.2353 (+)
Model 3	1	0.94 (0.80–1.16)	0.95 (0.89–0.99)	0.80 (0.62–0.91)	1.02 (0.69–1.24)	0.3265 (+)
Model 4	1	0.97 (0.82–1.12)	0.93 (0.74–1.14)	0.75 (0.61–0.92)	1.02 (0.76–1.32)	0.4086 (−)

^1)^ *p*-value by χ^2^ (chi-square) test. Model 1: crude, model 2: age, model 3: age and BMI, and model 4: age, BMI, physical activity, and smoking.

**Table 3:** Association between calcium intake quintiles and the prevalence of osteoporosis as determined by bone mineral density.

	Q1	Q2	Q3	Q4	Q5	*p* for Trend
Total	n = 1454	n = 1455	n = 1455	n = 1455	n = 1454	
Age (mean, SE)	57.28 ± 0.86	50.51 ± 0.73	48.65 ± 0.63	47.33 ± 0.60	47.24 ± 0.55	<0.0001 (−)
Vitamin D (ng/mL)	17.44 ± 0.33	17.88 ± 0.32	18.18 ± 0.33	18.38 ± 0.28	18.70 ± 0.29	0.0001 (+)
Ca Intake (mg/day)						
Mean, SE	153.55 ± 1.57	280.97 ± 1.11	401.55 ± 1.36	562.74 ± 1.99	980.06 ± 12.73	<0.0001 (+)
Median, SE	159.71 ± 1.63	282.01 ± 1.90	400.91 ± 1.72	559.11 ± 3.15	868.61 ± 11.36	-
Intake range	19.29–223.82	223.88–334.84	334.88–470.12	470.15–677.51	678.43–5047.19	-
Prevalence of Osteoporosis						
Normal, n (%)	1054 (77.20)	1248 (89.17)	1297 (92.71)	1343 (95.45)	1354 (95.67)	<0.0001 ^1)^
Osteoporosis, n (%)	400 (22.80)	207 (10.83)	158 (7.29)	112 (4.55)	100 (4.33)	
Odds Ratio (95% Confidence Interval)						
Model 1	1	0.37 (0.29–0.58)	0.27 (0.14–0.38)	0.155 (0.13–0.21)	0.14 (0.06–0.22)	<0.0001 (−)
Model 2	1	0.68 (0.51–0.97)	0.57 (0.40–0.87)	0.345 (0.18–0.50)	0.59 (0.38–0.86)	<0.0001 (−)
Model 3	1	0.66 (0.60–1.00)	0.67 (0.48–0.93)	0.564 (0.32–0.73)	0.61 (0.38–0.93)	0.0015 (−)
Model 4	1	0.63 (0.56–0.92)	0.66 (0.43–1.01)	0.44 (0.24–0.82)	0.63 (0.45–0.97)	0.0023 (−)
Male						
Age (mean, SE)	53.24 ± 1.26	50.30 ± 0.99	48.21 ± 0.86	47.30 ± 0.70	47.18 ± 0.69	<0.0001 (−)
Vitamin D (ng/mL)	19.32 ± 0.47	19.49 ± 0.43	19.64 ± 0.40	19.29 ± 0.38	19.45 ± 0.36	0.0001 (+)
Ca Intake (mg/day)						
Mean, SE	192.05 ± 2.96	335.80 ± 1.72	465.25 ± 1.89	640.58 ± 2.89	1075.46 ± 18.17	0.9898 (−)
Median, SE	203.87 ± 5.74	333.60 ± 2.90	464.08 ± 2.81	640.91 ± 5.24	956.86 ± 12.94	-
Intake range	34.36–269.18	269.52–398.38	398.66–533.66	533.77–754.16	754.30–5047.19	-
Prevalence of Osteoporosis						
Normal, n (%)	611 (92.62)	646 (96.87)	655 (98.02)	661 (98.80)	661 (98.42)	<0.0001 ^1)^
Osteoporosis, n (%)	65 (7.38)	31 (3.13)	22 (1.98)	16 (1.20)	15 (1.58)	
Odds Ratio (95% Confidence Interval)						
Model 1	1	0.36 (0.20–0.62)	0.35 (0.13–0.49)	0.14 (0.06–0.27)	0.12 (0.05–0.42)	<0.0001 (−)
Model 2	1	0.64 (0.31–0.92)	0.55 (0.14–1.06)	0.27 (0.06–0.96)	0.58 (0.21–1.11)	0.0074 (−)
Model 3	1	0.63 (0.34–1.15)	0.54 (0.31–1.13)	0.43 (0.32–1.07)	0.53 (0.27–1.57)	0.0562 (−)
Model 4	1	0.56 (0.30–1.06)	0.51 (0.27–1.09	0.52 (0.12–1.15)	0.49 (0.27–1.50)	0.0819 (−)
Female						
Age (mean, SE)	58.52 ± 1.03	52.33 ± 0.94	49.27 ± 0.84	47.38 ± 0.85	47.27 ± 0.76	<0.0001 (−)
Vitamin D (ng/mL)	16.32 ± 0.39	16.64 ± 0.36	16.52 ± 0.41	16.54 ± 0.33	17.05 ± 0.33	0.0001 (+)
Ca Intake (mg/day)						
Mean, SE	134.12 ± 1.97	244.50 ± 1.32	349.51 ± 1.65	494.03 ± 2.51	887.44 ± 17.32	0.1494
Median, SE	139.26 ± 1.89	243.75 ± 2.67	350.09 ± 2.28	493.99 ± 3.97	788.96 ± 12.95	-
Intake range	19.29–193.62	193.70–293.09	293.17–407.24	407.33–595.09	595.56–4096.11	-
Prevalence of Osteoporosis						
Normal, n (%)	512 (71.32)	581 (78.00)	637 (85.16)	653 (88.89)	679 (90.64)	<0.0001 ^1)^
Osteoporosis, n (%)	266 (28.68)	197 (22.00)	141 (14.84)	125 (11.11)	99 (9.36)	
Odds Ratio (95% Confidence Interval)						
Model 1	1	0.71 (0.43–0.94)	0.443 (0.320–0.61)	0.33 (0.22–0.52)	0.25 (0.18–0.56)	<0.0001 (−)
Model 2	1	0.95 (0.83–1.00)	0.816 (0.678–1.00)	0.70 (0.43–0.92)	0.64 (0.27–0.97)	<0.0001 (−)
Model 3	1	0.94 (0.75–1.93)	0.942 (0.562–1.59)	0.66 (0.36–0.91)	0.54 (0.39–1.19)	0.0415 (−)
Model 4	1	0.91 (0.70–1.91)	0.934 (0.554–1.60)	0.55 (0.18–0.91)	0.53 (0.37–1.19)	0.0746 (−)

^1)^ *p*-value by χ^2^ (chi-square) test. Model 1: crude, model 2: age, model 3: age and BMI, and model 4: age, BMI, physical activity, and smoking.

## Data Availability

The original contributions presented in the study are included in the article; further inquiries can be directed to the corresponding author.
